# Supplementary material for: Inhibition of miR-96-5p in the mouse brain increases glutathione levels by altering NOVA1 expression
Source: Commun Biol. 2021 Feb 10;4:182. doi: 10.1038/s42003-021-01706-0 (PMC7876013; doi:10.1038/s42003-021-01706-0)
Supplement: Supplementary file 1 — Supplementary Information [file 42003_2021_1706_MOESM1_ESM.pdf]

Supplementary Information for:

Inhibition of miR-96-5p in the mouse brain increases glutathione levels by altering NOVA1 expression

Authors: Chisato Kinoshita[1], Kazue Kikuchi-Utsumi[1], Koji Aoyama[1], Ryo Suzuki[2], Yayoi Okamoto[1,3], Nobuko Matsumura[1], Daiki Omata[2], Kazuo Maruyama[4] and Toshio Nakaki[1,5,*]

Affiliation: [1]Department of Pharmacology, Teikyo University School of Medicine, 2-11-1 Kaga, Itabashi, Tokyo 173-8605, Japan

[2]Laboratory of Drug and Gene Delivery, Faculty of Pharma-Science, Teikyo University, 2-11-1 Kaga, Itabashi, Tokyo 173-8605, Japan

[3]Teikyo University Support Center for Women Physicians and Researchers, 2-11-1 Kaga, Itabashi, Tokyo 173-8605, Japan

[4]Laboratory of Theranostics, Faculty of Pharma-Science, Teikyo University, 2-11-1 Kaga, Itabashi, Tokyo 173-8605, Japan

[5]Faculty of Pharma-Science, Teikyo University, 2-11-1 Kaga, Itabashi, Tokyo 173-8605, Japan

*Corresponding author: Toshio Nakaki

Tel: +81-3-3964-1211 (ext: 45203)

Fax: +81-3-3964-0602

E-mail: nakaki@med.teikyo-u.ac.jp

Supplementary Table 1

Gel	MASCOT MS/MS ion-serch result									miR-96-5p target prediction	
Band size	Accession	Description	Score	Coverage	# Peptides	# AAs	MW [kDa]	calc. pI	P value	mirSVR score	PhastCons Score
43.1 kDa	Q99L45	Eukaryotic translation initiation factor 2 subunit 2 OS=Mus musculus GN=Eif2s2 PE=1 SV=1 - [IF2B_MOUSE]	1134.22	49.85	18	331	38.1	5.80	3.78E-114	-0.1019	0.7071
	O70172	Phosphatidylinositol 5-phosphate 4-kinase type-2 alpha OS=Mus musculus GN=Pip4k2a PE=1 SV=1 - [PI42A_MOUSE]	387.01	23.70	10	405	46.1	6.99	1.99E-39	-0.0075	0.5514
	Q60668	Heterogeneous nuclear ribonucleoprotein D0 OS=Mus musculus GN=Hnrnpd PE=1 SV=2 - [HNRPD_MOUSE]	329.31	25.07	8	355	38.3	7.81	1.17E-33	-0.0022	0.6743
	Q9JKN6	RNA-binding protein Nova-1 OS=Mus musculus GN=Nova1 PE=1 SV=2 - [NOVA1_MOUSE]	100.75	7.10	3	507	51.7	8.72	8.42E-11	-1.2513	0.8601
										-0.7122	0.8601
	Q9QYR6	Microtubule-associated protein 1A OS=Mus musculus GN=Map1a PE=1 SV=2 - [MAP1A_MOUSE]	93.69	1.22	3	2776	300.0	5.00	4.27E-10	-0.0043	0.5447
										-0.0031	0.5977
	Q9WV60	Glycogen synthase kinase-3 beta OS=Mus musculus GN=Gsk3b PE=1 SV=2 - [GSK3B_MOUSE]	69.32	6.90	3	420	46.7	8.78	1.17E-07	-0.0003	0.5645
	A2A935	PR domain zinc finger protein 16 OS=Mus musculus GN=Prdm16 PE=1 SV=1 - [PRD16_MOUSE]	63.19	0.71	1	1275	140.8	6.10	4.80E-07	-0.1834	0.6619
	P20357	Microtubule-associated protein 2 OS=Mus musculus GN=Map2 PE=1 SV=2 - [MTAP2_MOUSE]	59.31	1.97	3	1828	199.0	4.91	1.17E-06	-0.0192	0.5153
										-0.0231	0.5813
	O55131	Septin-7 OS=Mus musculus GN=Sept7 PE=1 SV=1 - [SEPT7_MOUSE]	52.35	4.36	2	436	50.5	8.57	5.82E-06	-0.9434	0.7536
	P61164	Alpha-centractin OS=Mus musculus GN=Actr1a PE=2 SV=1 - [ACTZ_MOUSE]	31.65	3.99	2	376	42.6	6.64	6.83E-04	-0.2171	0.5431
51.3 kDa	Q9JKN6	RNA-binding protein Nova-1 OS=Mus musculus GN=Nova1 PE=1 SV=2 - [NOVA1_MOUSE]	498.27	23.27	13	507	51.7	8.72	1.49E-50	-1.2513	0.8601
										-0.7122	0.8601
	P62814	V-type proton ATPase subunit B, brain isoform OS=Mus musculus GN=Atp6v1b2 PE=1 SV=1 - [VATB2_MOUSE]	225.71	10.18	6	511	56.5	5.81	2.68E-23	-0.1299	0.7481
	Q8VIJ6	Splicing factor, proline- and glutamine-rich OS=Mus musculus GN=Sfpq PE=1 SV=1 - [SFPQ_MOUSE]	221.46	4.43	3	699	75.4	9.44	7.15E-23	-0.0465	0.6582
	Q8VD63	Testis-specific Y-encoded-like protein 4 OS=Mus musculus GN=Tspyl4 PE=2 SV=1 - [TSYL4_MOUSE]	180.92	14.04	5	406	44.8	6.99	8.09E-19	-0.0096	0.6495
										-0.0087	0.6623
	Q9WUM4	Coronin-1C OS=Mus musculus GN=Coro1c PE=1 SV=2 - [COR1C_MOUSE]	179.11	12.66	6	474	53.1	7.08	1.23E-18	-0.0778	0.6578
	P56959	RNA-binding protein FUS OS=Mus musculus GN=Fus PE=2 SV=1 - [FUS_MOUSE]	157.83	8.69	3	518	52.6	9.36	1.65E-16	-0.0622	0.5644
	P68368	Tubulin alpha-4A chain OS=Mus musculus GN=Tuba4a PE=1 SV=1 - [TBA4A_MOUSE]	149.21	15.18	7	448	49.9	5.06	1.20E-15	-0.0033	0.5957
	Q6PHZ2	Calcium/calmodulin-dependent protein kinase type II subunit delta OS=Mus musculus GN=Camk2d PE=1 SV=1 - [KCC2D_MOUSE]	146.93	8.42	4	499	56.3	7.25	2.03E-15	-0.0144	0.5589
										-0.0215	0.7159
										-0.0024	0.5452
	Q923T9	Calcium/calmodulin-dependent protein kinase type II subunit gamma OS=Mus musculus GN=Camk2g PE=1 SV=1 - [KCC2G_MOUSE]	142.52	12.10	6	529	59.6	7.58	5.60E-15	-0.0016	0.6328
	O54724	Polymerase I and transcript release factor OS=Mus musculus GN=Ptrf PE=1 SV=1 - [PTRF_MOUSE]	125.91	11.73	4	392	43.9	5.52	2.57E-13	-0.0039	0.6006
	P27546	Microtubule-associated protein 4 OS=Mus musculus GN=Map4 PE=1 SV=3 - [MAP4_MOUSE]	63.44	2.31	2	1125	117.4	4.98	4.53E-07	-0.0013	0.4873
	P20357	Microtubule-associated protein 2 OS=Mus musculus GN=Map2 PE=1 SV=2 - [MTAP2_MOUSE]	58.46	0.88	1	1828	199.0	4.91	1.43E-06	-0.0192	0.5153
										-0.0231	0.5813
	Q8C8N2	Protein SCAI OS=Mus musculus GN=Scai PE=1 SV=2 - [SCAI_MOUSE]	56.92	3.14	2	606	70.2	8.60	2.03E-06	-0.0035	0.6005
										-0.0056	0.57
	Q9QYR6	Microtubule-associated protein 1A OS=Mus musculus GN=Map1a PE=1 SV=2 - [MAP1A_MOUSE]	55.09	0.83	2	2776	300.0	5.00	3.10E-06	-0.0043	0.5447
										-0.0031	0.5977
	Q9EQ20	Methylmalonate-semialdehyde dehydrogenase [acylating], mitochondrial OS=Mus musculus GN=Aldh6a1 PE=1 SV=1 - [MMSA_MOUSE]	40.42	1.87	1	535	57.9	8.07	9.08E-05	-0.1313	0.5553
	O08739	AMP deaminase 3 OS=Mus musculus GN=Ampd3 PE=2 SV=2 - [AMPD3_MOUSE]	37.11	1.17	1	766	88.6	7.33	1.95E-04	-0.0078	0.5876
	O35841	Apoptosis inhibitor 5 OS=Mus musculus GN=Api5 PE=1 SV=2 - [API5_MOUSE]	29.79	1.79	1	504	56.7	5.92	1.05E-03	-0.8038	0.7431
										-0.0168	0.5994
	P10637	Microtubule-associated protein tau OS=Mus musculus GN=Mapt PE=1 SV=3 - [TAU_MOUSE]	29.10	2.05	1	733	76.2	6.79	1.23E-03	-0.0002	0.5349
										-0.0279	0.5441
										-0.0206	0.531
	Q8BTV2	Cleavage and polyadenylation specificity factor subunit 7 OS=Mus musculus GN=Cpsf7 PE=1 SV=2 - [CPSF7_MOUSE]	22.20	1.70	1	471	52.0	8.00	6.03E-03	-0.7961	0.6897
80.7 kDa	O08739	AMP deaminase 3 OS=Mus musculus GN=Ampd3 PE=2 SV=2 - [AMPD3_MOUSE]	1128.09	26.37	22	766	88.6	7.33	1.55E-113	-0.0078	0.5876
	Q8BZ98	Dynamin-3 OS=Mus musculus GN=Dnm3 PE=1 SV=1 - [DYN3_MOUSE]	458.59	12.98	12	863	97.1	8.35	1.38E-46	-0.0012	0.5933
	Q8VIJ6	Splicing factor, proline- and glutamine-rich OS=Mus musculus GN=Sfpq PE=1 SV=1 - [SFPQ_MOUSE]	202.04	12.59	8	699	75.4	9.44	6.25E-21	-0.0465	0.6582
	Q03141	MAP/microtubule affinity-regulating kinase 3 OS=Mus musculus GN=Mark3 PE=1 SV=2 - [MARK3_MOUSE]	197.80	10.62	7	753	84.3	9.51	1.66E-20	-0.0828	0.6587
	Q05512	Serine/threonine-protein kinase MARK2 OS=Mus musculus GN=Mark2 PE=1 SV=3 - [MARK2_MOUSE]	106.65	3.74	2	776	86.3	9.67	2.16E-11	-0.0024	0.5652
	Q9Z268	RasGAP-activating-like protein 1 OS=Mus musculus GN=Rasal1 PE=2 SV=2 - [RASL1_MOUSE]	101.18	3.75	3	799	89.3	6.37	7.62E-11	-0.0025	0.5704
	Q9DBG3	AP-2 complex subunit beta OS=Mus musculus GN=Ap2b1 PE=1 SV=1 - [AP2B1_MOUSE]	69.16	4.16	4	937	104.5	5.38	1.21E-07	-0.2615	0.644
										-0.001	0.5424
	P28740	Kinesin-like protein KIF2A OS=Mus musculus GN=Kif2a PE=1 SV=2 - [KIF2A_MOUSE]	57.98	3.12	3	705	79.7	6.73	1.59E-06	-0.1331	0.7069
										-0.163	0.7069
	Q9WV92	Band 4.1-like protein 3 OS=Mus musculus GN=Epb41l3 PE=1 SV=1 - [E41L3_MOUSE]	47.92	1.83	2	929	103.3	5.31	1.61E-05	-1.1251	0.6907
	Q68FF6	ARF GTPase-activating protein GIT1 OS=Mus musculus GN=Git1 PE=1 SV=1 - [GIT1_MOUSE]	39.76	1.17	1	770	85.2	6.93	1.06E-04	-0.0028	0.5952
										-0.1405	0.6964
87.6 kDa	Q8VIJ6	Splicing factor, proline- and glutamine-rich OS=Mus musculus GN=Sfpq PE=1 SV=1 - [SFPQ_MOUSE]	5877.73	35.34	25	699	75.4	9.44	0.00E+00	-0.0465	0.6582
	O35066	Kinesin-like protein KIF3C OS=Mus musculus GN=Kif3c PE=1 SV=3 - [KIF3C_MOUSE]	1085.44	30.65	23	796	89.9	8.06	2.86E-109	-0.0494	0.5008
	P28740	Kinesin-like protein KIF2A OS=Mus musculus GN=Kif2a PE=1 SV=2 - [KIF2A_MOUSE]	1073.71	28.09	22	705	79.7	6.73	4.26E-108	-0.1331	0.7069
										-0.163	0.7069
	O70318	Band 4.1-like protein 2 OS=Mus musculus GN=Epb41l2 PE=1 SV=2 - [E41L2_MOUSE]	747.31	21.86	20	988	109.9	5.43	1.86E-75	-0.1136	0.7264
	Q9WV92	Band 4.1-like protein 3 OS=Mus musculus GN=Epb41l3 PE=1 SV=1 - [E41L3_MOUSE]	119.74	4.20	4	929	103.3	5.31	1.06E-12	-1.1251	0.6907
	Q8BXK8	Arf-GAP with GTPase, ANK repeat and PH domain-containing protein 1 OS=Mus musculus GN=Agap1 PE=2 SV=1 - [AGAP1_MOUSE]	113.46	4.43	4	857	94.4	7.94	4.51E-12	-0.0001	0.5172
										-0.0002	0.5317
	Q9Z2H5	Band 4.1-like protein 1 OS=Mus musculus GN=Epb41l1 PE=1 SV=2 - [E41L1_MOUSE]	69.97	2.50	2	879	98.3	5.62	1.01E-07	-0.0054	0.6166
										-0.0068	0.7237
	Q9DBG3	AP-2 complex subunit beta OS=Mus musculus GN=Ap2b1 PE=1 SV=1 - [AP2B1_MOUSE]	50.28	1.49	2	937	104.5	5.38	9.38E-06	-0.2615	0.644
										-0.001	0.5424
89.2 kDa	Q8VIJ6	Splicing factor, proline- and glutamine-rich OS=Mus musculus GN=Sfpq PE=1 SV=1 - [SFPQ_MOUSE]	2121.94	31.76	23	699	75.4	9.44	6.39E-213	-0.0465	0.6582
	P28740	Kinesin-like protein KIF2A OS=Mus musculus GN=Kif2a PE=1 SV=2 - [KIF2A_MOUSE]	1538.64	30.07	24	705	79.7	6.73	1.37E-154	-0.1331	0.7069
										-0.163	0.7069
	Q9DBG3	AP-2 complex subunit beta OS=Mus musculus GN=Ap2b1 PE=1 SV=1 - [AP2B1_MOUSE]	190.98	10.14	10	937	104.5	5.38	7.97E-20	-0.2615	0.644
										-0.001	0.5424
	Q9QYR6	Microtubule-associated protein 1A OS=Mus musculus GN=Map1a PE=1 SV=2 - [MAP1A_MOUSE]	190.66	2.23	6	2776	300.0	5.00	8.59E-20	-0.0043	0.5447
										-0.0031	0.5977
	Q8BXK8	Arf-GAP with GTPase, ANK repeat and PH domain-containing protein 1 OS=Mus musculus GN=Agap1 PE=2 SV=1 - [AGAP1_MOUSE]	114.79	4.43	4	857	94.4	7.94	3.32E-12	-0.0001	0.5172
										-0.0002	0.5317
	P58069	Ras GTPase-activating protein 2 OS=Mus musculus GN=Rasa2 PE=1 SV=2 - [RASA2_MOUSE]	101.41	3.19	3	847	96.3	7.59	7.23E-11	-0.006	0.6136
										-0.0376	0.5775
	P97386	DNA ligase 3 OS=Mus musculus GN=Lig3 PE=1 SV=2 - [DNLI3_MOUSE]	89.22	3.74	4	1015	113.0	8.98	1.20E-09	-0.0005	0.3903
	Q62448	Eukaryotic translation initiation factor 4 gamma 2 OS=Mus musculus GN=Eif4g2 PE=1 SV=2 - [IF4G2_MOUSE]	49.00	1.88	2	906	102.0	7.14	1.26E-05	-0.2242	0.7629
	Q9Z2H5	Band 4.1-like protein 1 OS=Mus musculus GN=Epb41l1 PE=1 SV=2 - [E41L1_MOUSE]	48.11	1.14	1	879	98.3	5.62	1.55E-05	-0.0054	0.6166
										-0.0068	0.7237
	O70318	Band 4.1-like protein 2 OS=Mus musculus GN=Epb41l2 PE=1 SV=2 - [E41L2_MOUSE]	43.50	2.23	2	988	109.9	5.43	4.47E-05	-0.1136	0.7264

List of binding protein candidates for GTRAP3-18 3’-UTR analyzed by nano-LC MS/MS ion-search, which are also analyzed by miRNA prediction algorithm for miR-96-5p targeting.

Supplementary Table 2

miRNA mimic	Sequence
negative control	5’-UCACCGGGUGUAAAUCAGCUUG-3’
miR-96-5p	5’-UUUGGCACUAGCACAUUUUUGCU-3’

miRNA inhibitor	Sequence
negative control	5’-TAACACGTCTATACGCCCA-3’
miR-96-5p	5’-GCAAAAATGTGCTAGTGCCAA-3’

Cloning of 3'-UTR		Primer sequence
GTRAP3-18	Forward	5’-GAGCTCACATAACTTACCTGAGCTAGG-3’
	Reverse	5’-ACGCGTAAATAAAGTCTCACC-3’
NOVA1	Forward	5’-GAGCTCGTGCCCCAGTTACACATCAGA-3’
	Reverse	5’-GCCGGCTGATGCTACATGATGAACTA-3’

Mutagenesis		Primer sequence
GTRAP3-18 Ptbp1-mut	Forward	5’- TCTTCTTGA CTTCTCAGACATGGTCTAGAATC -3’
	Reverse	5’-GACAAGTCAAGAAGATATCACTGTGCTAAAGA-3’
GTRAP3-18 unbound-mut	Forward	5’-TGTCGTTGACTTGTCAGACATGGTCTAGAATC -3’
	Reverse	5’-GACAAGTCAACGACATATCACTGTGCTAAAGA-3’
GTRAP3-18 deletion-mut	Forward	5’-CAGTGATAGACTTCACAGACATGGTC -3’
	Reverse	5’-GTGAAGTCTATCACTGTGCTAAAGATC-3’
NOVA1 mut1	Forward	5’-GAAAGTCGGAACAAATTATTGATAGCT-3’
	Reverse	5’-TTTGTTCCGACTTTCACTTTTGTTTAT-3’
NOVA1 mut2	Forward	5’-CTGAGTCGGAACTGTCCAGGCCATTTG-3’
	Reverse	5’-ACAGTTCCGACTCAGGAGAGGTACAGA-3’

quantified RT-PCR		Primer sequence
GTRAP3-18	Forward	5’- GGAACAACCGTGTAGTGAGCAA -3’
	Reverse	5’- TGATGCCGAACACAAAGACC -3’
NOVA1	Forward	5’-AGTATCCTACAACCTCAG -3’
	Reverse	5’- CTCCATTATAGCCTTCAC-3’
GAPDH	Forward	5’- AAAATGGTGAAGGTCGGTGTG -3’
	Reverse	5’- AATGAAGGGGTCGTTGATGG -3’

RNA synthesis		Primer sequence
GTRAP3-18	Forward	5’-TAATACGACTCACTATAGGGAGAACATAACTTACCTGA-3’
	Reverse	5’-GGATCACTAGTAAGCTTAGATCTTAAATAAAGTCTCACC-3’

List of miRNA mimic, inhibitor and primer sequence used in the manuscript

Supplementary Figure 1

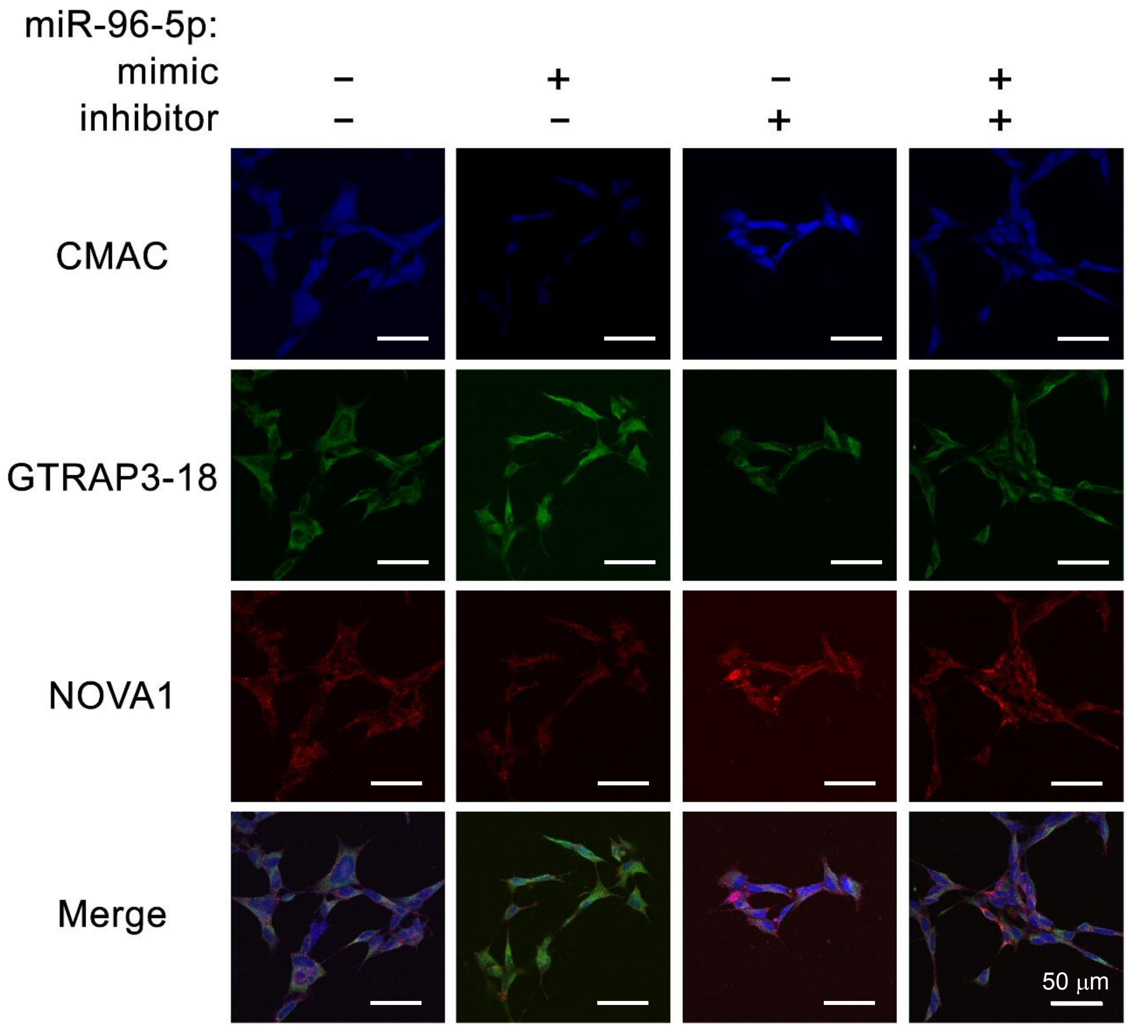

The effect of miR-96-5p mimic and/or inhibitor transfection in SH-SY5Y cells
Confocal images show the effect of miR-96-5p mimic and/or inhibitor transfection on the intensity of CMAC as a marker of GSH (blue), the expressions of GTRAP3-18 (green) and NOVA1 (red). Scale bar, 50μm.

Supplementary Figure 2

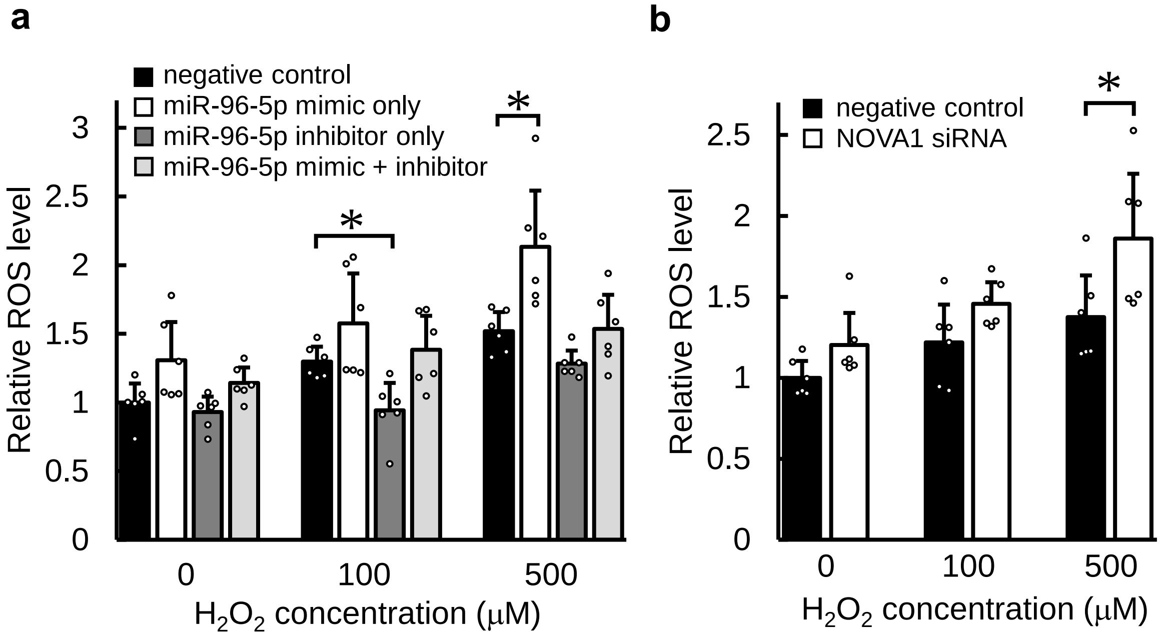

The effect of miR-96-5p mimic and/or inhibitor transfection on ROS level in Neuro2a cells

a Relative ROS levels in Neuro2a cells transfected with appropriate combination of miR-96-5p mimic and/or inhibitor are shown. Data represent mean values ± SD obtained from six independent experiments and were plotted as individual data points from each experiment. Data were analyzed by one-way ANOVA ($F(3,20)$=5.23, p=0.0079 for 100 μM H_2O_2 treatment, $F(3,20)$=9.21, p= 0.00050 for 500 μM H_2O_2 treatment)and Tukey's HSD test. *p<0.05 relative to the negative control in each H_2O_2 concentration. **b** Relative ROS levels in Neuro2a cells transfected with NOVA1 siRNA or negative control are shown. Data represent mean values ± SD obtained from six independent experiments and were plotted as individual data points from each experiment. Data were analyzed by Student's t-test, two-sided. *p<0.05 relative to the negative control in each H_2O_2 concentration.

Supplementary Figure 3

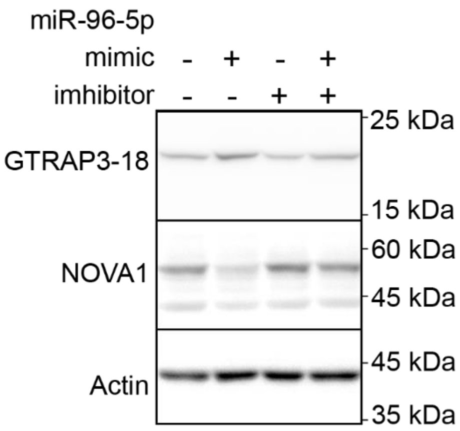

The effect of miR-96-5p on the expressions of GTRAP3-18 and NOVA1 in HEK293 cells

The endogenous expressions of GTRAP3-18, NOVA1 and β-actin in HEK293 cells with transfection of miR-96-5p mimic and/or inhibitor are shown. Molecular weight markers are depicted at right.

Supplementary Figure 4

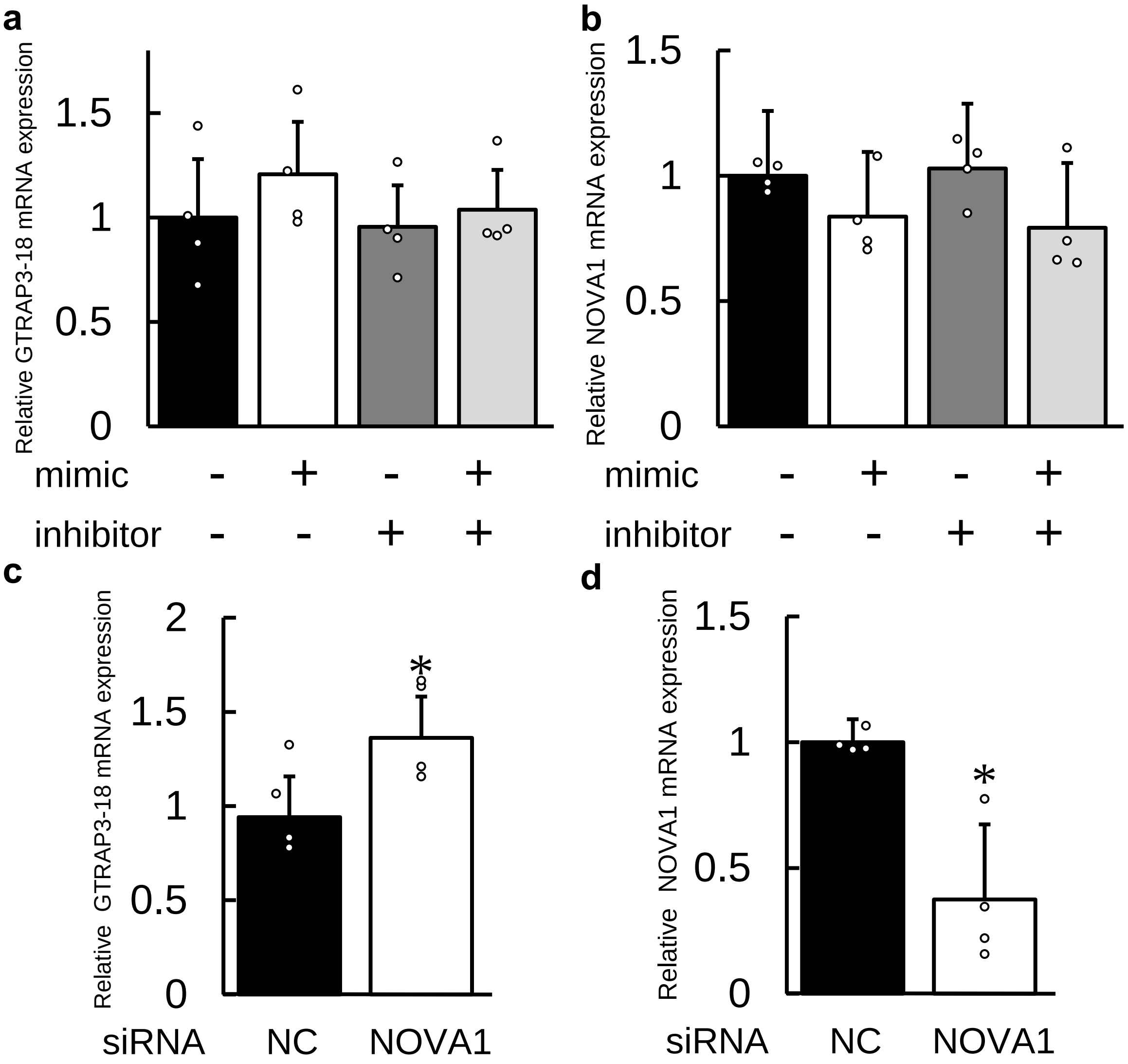

Changes in the mRNA expression of GTRAP3-18 and NOVA1 with manipulation of miR-96-5p or NOVA1 expression

a, b Relative mRNA expressions of GTRAP3-18 (panel **a**) or NOVA1 (panel **b**) with transfection of miR-96-5p mimic and/or inhibitor are shown. Data represent mean values ± SD obtained from four independent experiments and were plotted as individual data points from each experiment. Data were analyzed by one-way ANOVA (For GTRAP3-18 expression; $F(3,12)$=0.99, p= 0.43, For NOVA1 expression; $F(3,12)$=2.32, p= 0.13). **c, d** Relative mRNA expressions of GTRAP3-18 (panel **c**) or NOVA1 (panel **d**) with transfection of NOVA1 siRNA or negative control (NC) siRNA are shown. Data represent mean values ± SD obtained from four independent experiments and were plotted as individual data points from each experiment. Data were analyzed by Student's t-test, two-sided. *p<0.05 relative to the negative control.

Supplementary Figure 5

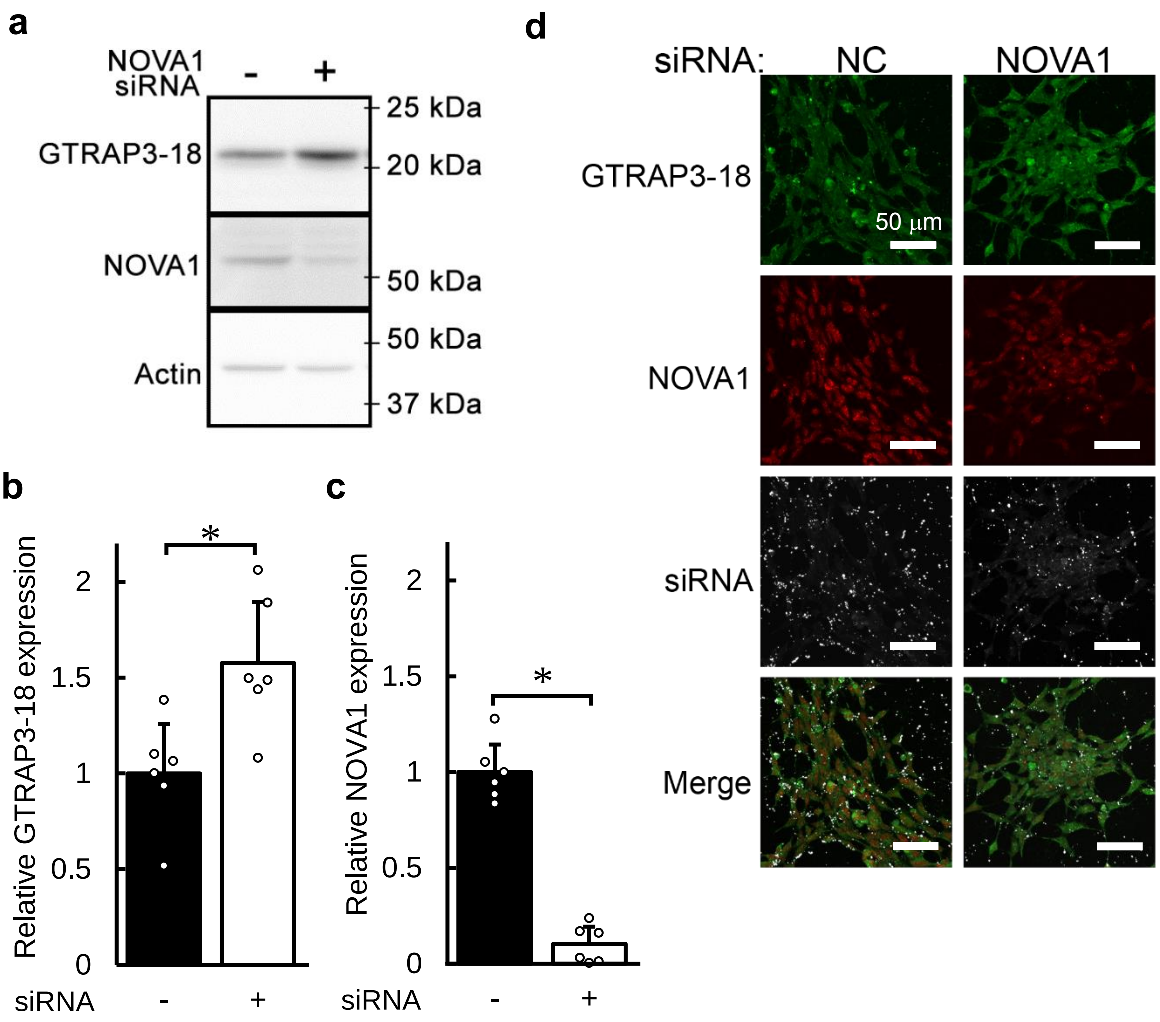

The effect of NOVA1 siRNA transfection in HEK293 and SH-SY5Y cells

a The endogenous expressions of GTRAP3-18, NOVA1 and β-actin after transfection of negative control (-) or NOVA1 siRNA (+) in HEK293 cells are shown. Molecular weight markers are depicted at right. **b, c** Quantification of the GTRAP3-18 (panel **b**) or NOVA1 (panel **c**) expression in panel **a** by densitometry. Data represent mean values ± SD obtained from six independent experiments and were plotted as individual data points from each experiment. Data were analyzed by Student's t-test, two-sided. *p<0.05 relative to the negative control. **d** Confocal images showing the effect of the NOVA1 siRNA or negative control (NC) transfection (white) on the intensity GTRAP3-18 (green) and NOVA1 (red). Scale bar, 50 μm.

Supplementary Figure 6

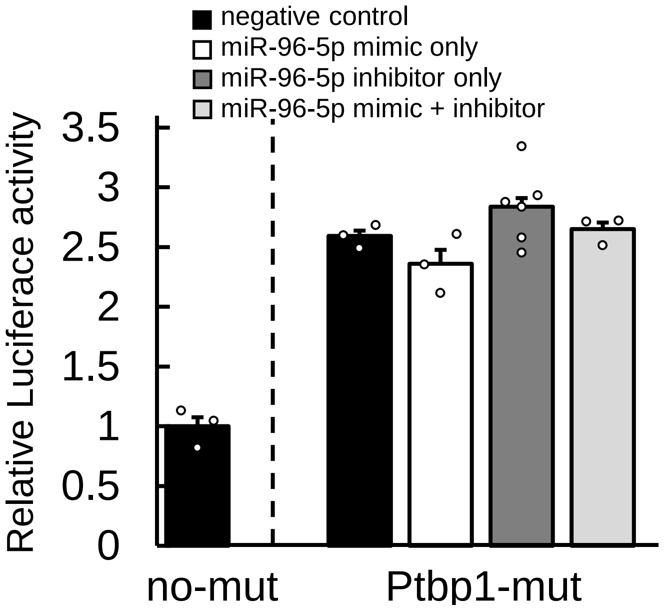

The effect of inserting mutation at predicted NOVA1 binding site on the 3'-UTR of GTRAP3-18.

Relative luciferase activity in SH-SY5Y cells transfected with the luciferase plasmids in Fig. 4d with negative control (*n*=3), miR-96-5p mimic only (*n*=3), miR-96-5p inhibitor only (*n*=6) or miR-96-5p mimic plus inhibitor (*n*=3) are shown. Data represent mean values ± SD and were plotted as individual data points. Data were analyzed by one-way ANOVA ($F(3,11)$=2.67, p= 0.099).

Supplementary Figure 7

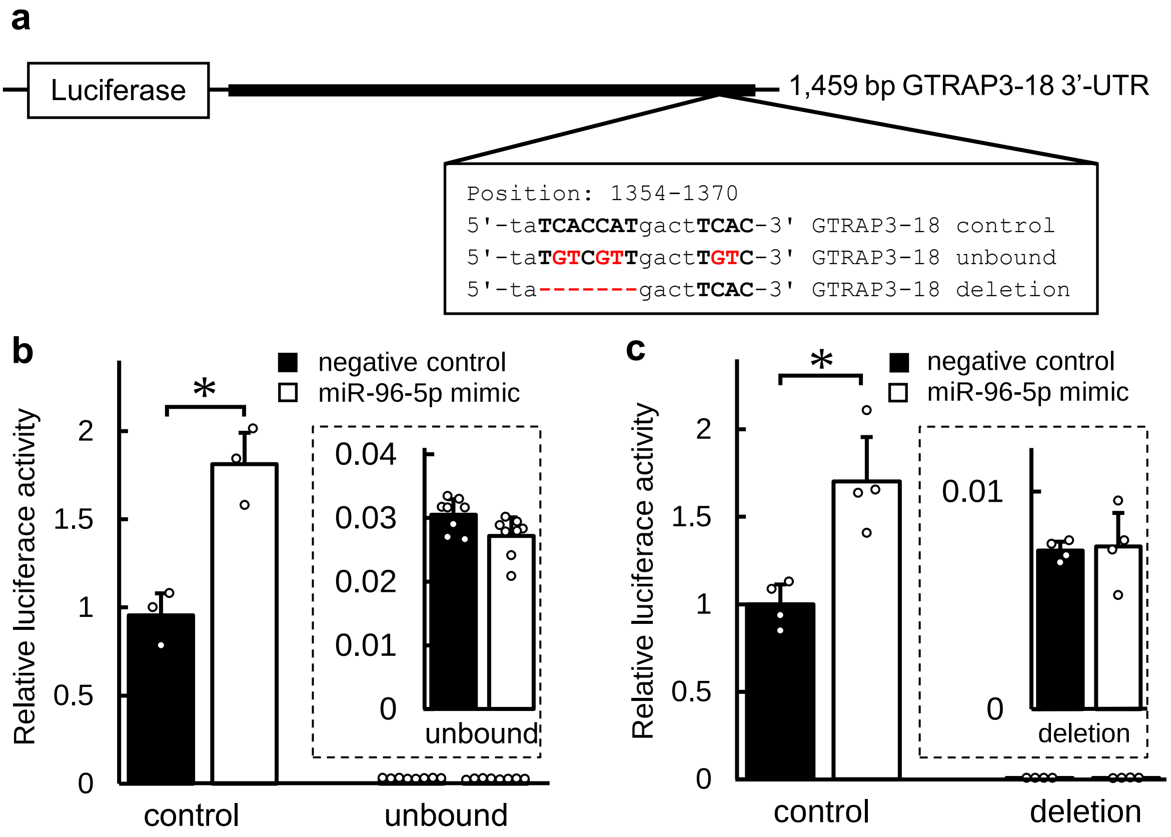

The effect of inserting point or deletion mutations at predicted NOVA1 binding site on the 3'-UTR of GTRAP3-18. a A schematic plot of the luciferase plasmids of GTRAP3-18 3'-UTR. The sequence for the predicted NOVA1 binding site is shown. A part of YCAY clusters (bold font) was mutated or deleted (red font). Constructs with either point mutant (unbound) or deletion mutant (deletion) were predicted not to be able to bind with any RBPs including NOVA1. **b** Relative luciferase activity in SH-SY5Y cells transfected with luciferase constructs in **a** with miR-96-5p mimic or negative control are shown. Data represent mean values ± SD obtained from independent samples (n=3 for control, n=8 for unbound mutant) and were plotted as individual data points. Data were analyzed by Student's t-test, two-sided. *p< 0.05 relative to negative control. Inset; enlargements of the graph using luciferase construct with unbound mutant. **c** Relative luciferase activity in SH-SY5Y cells transfected with luciferase constructs in **a** with miR-96-5p mimic or negative control are shown. Data represent mean values ± SD obtained from four independent experiments and were plotted as individual data points. Data were analyzed by Student's t-test, two-sided. *p< 0.05 relative to negative control. Inset; enlargements of the graph using luciferase construct with deletion mutant.

Supplementary Figure 8

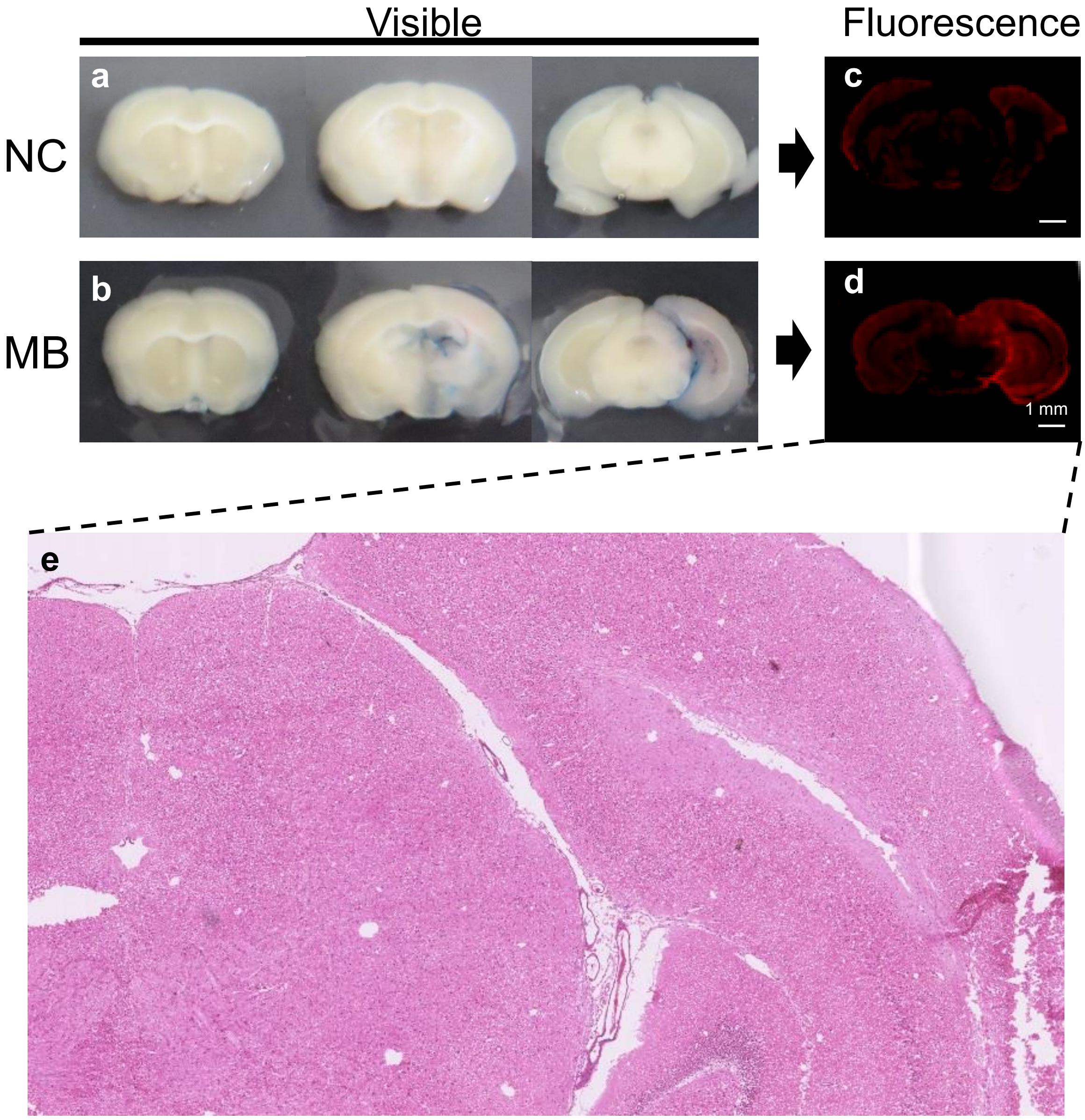

Blood-brain-barrier leakage of evans blue in the treated side of hippocampus

a-d Photographs of coronal brain slices after intra-arterial administration of evans blue with (MB) or without (NC) microbubble were shown. Panel **a** and **b** shows visual images of brain slice series. Panel **c** and **d** shows the evans blue fluorescence detection with 620 nm excitation and 680 nm emission. Scale bar, 1 mm. **e** Staining of coronal brain slice in panel **d** with hematoxylin and eosin showed histologically no hallmark of brain damage.

Supplementary Figure 9

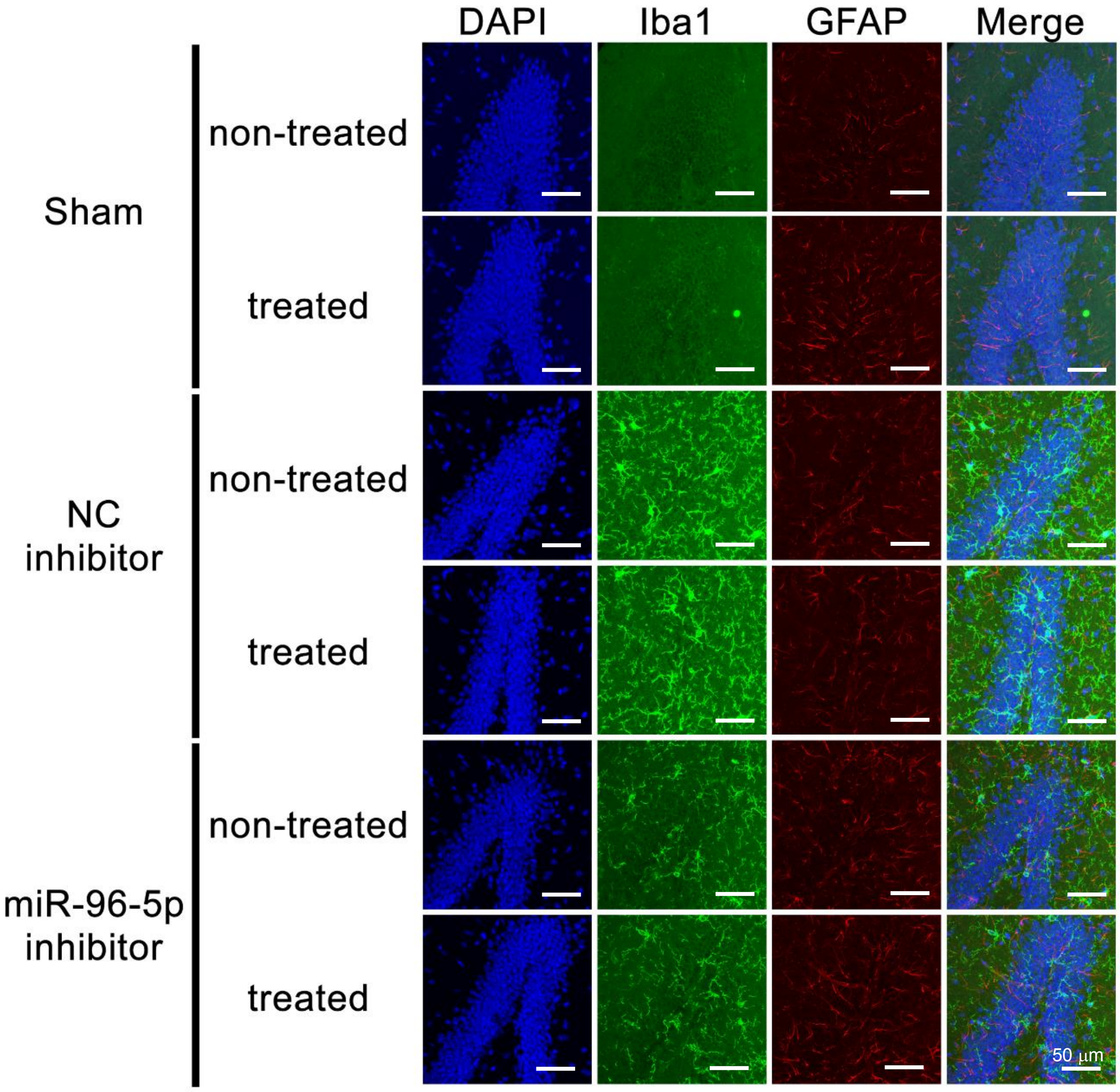

Effect of the intra-arterial administration of miR-96-5p inhibitor with microbubbles and ultrasound on microglial and glial activation. Confocal images show the effect of administration of miR-96-5p inhibitor or negative control (NC) inhibitor on the expressions of Iba1 (green) as microglial marker and GFAP (red) as a glial marker. The nuclei were stained with DAPI (blue). To see the effect of ultrasound (US), the result of sham operation without US application (sham) is also shown. Scale bar, 50 μm.

Supplementary Figure 10

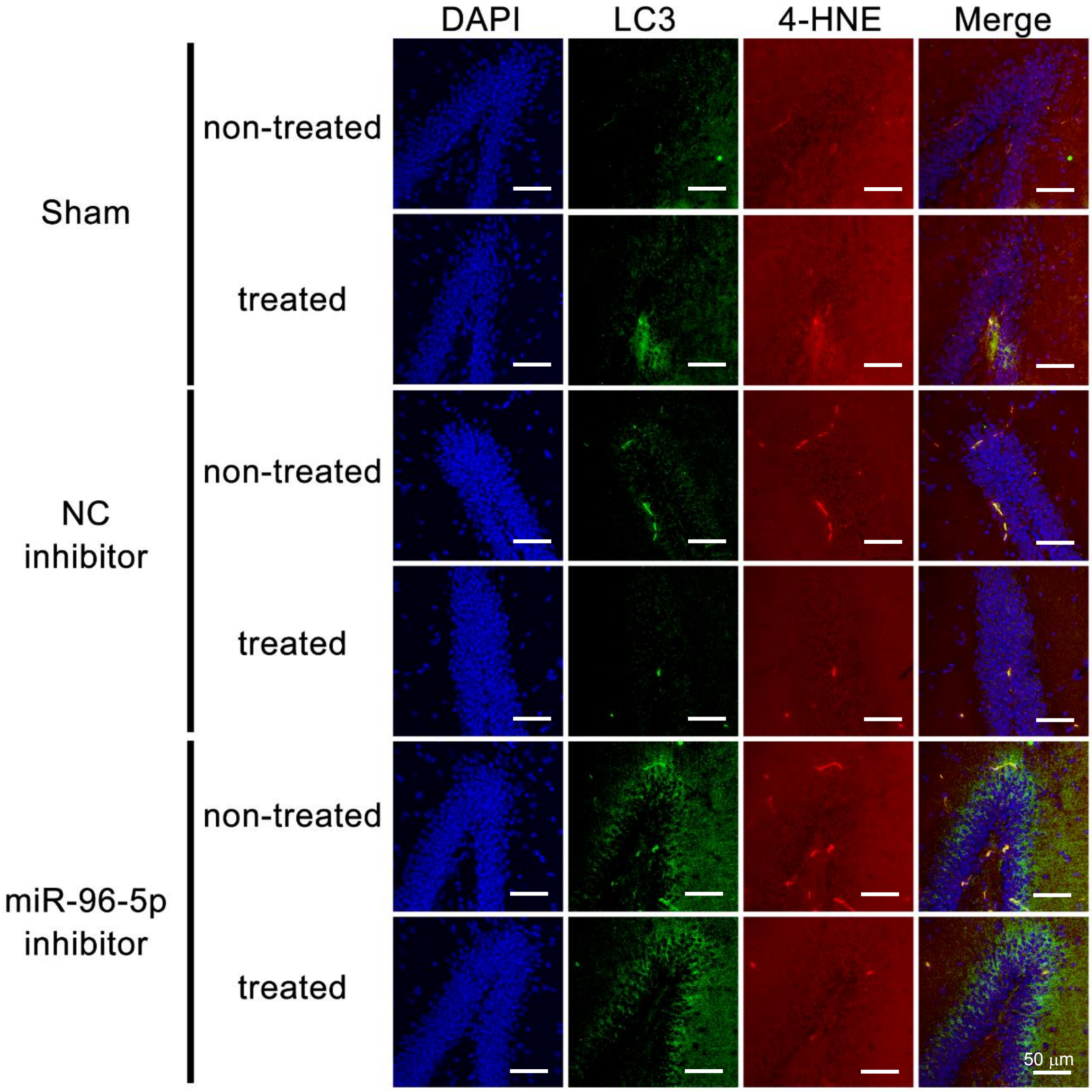

Effect of the intra-arterial administration of miR-96-5p inhibitor with microbubbles and ultrasound on autophagy activation and lipid oxidation. Confocal images show the effect of administration of miR-96-5p inhibitor or negative control (NC) inhibitor on the expressions of LC3 (green) as a marker of autophagy and 4-HNE (red) as a marker of lipid oxidation. The nuclei were stained with DAPI (blue). To see the effect of ultrasound (US), the result of sham operation without US application (sham) is also shown. Scale bar, 50 μm.

Supplementary Figure 11

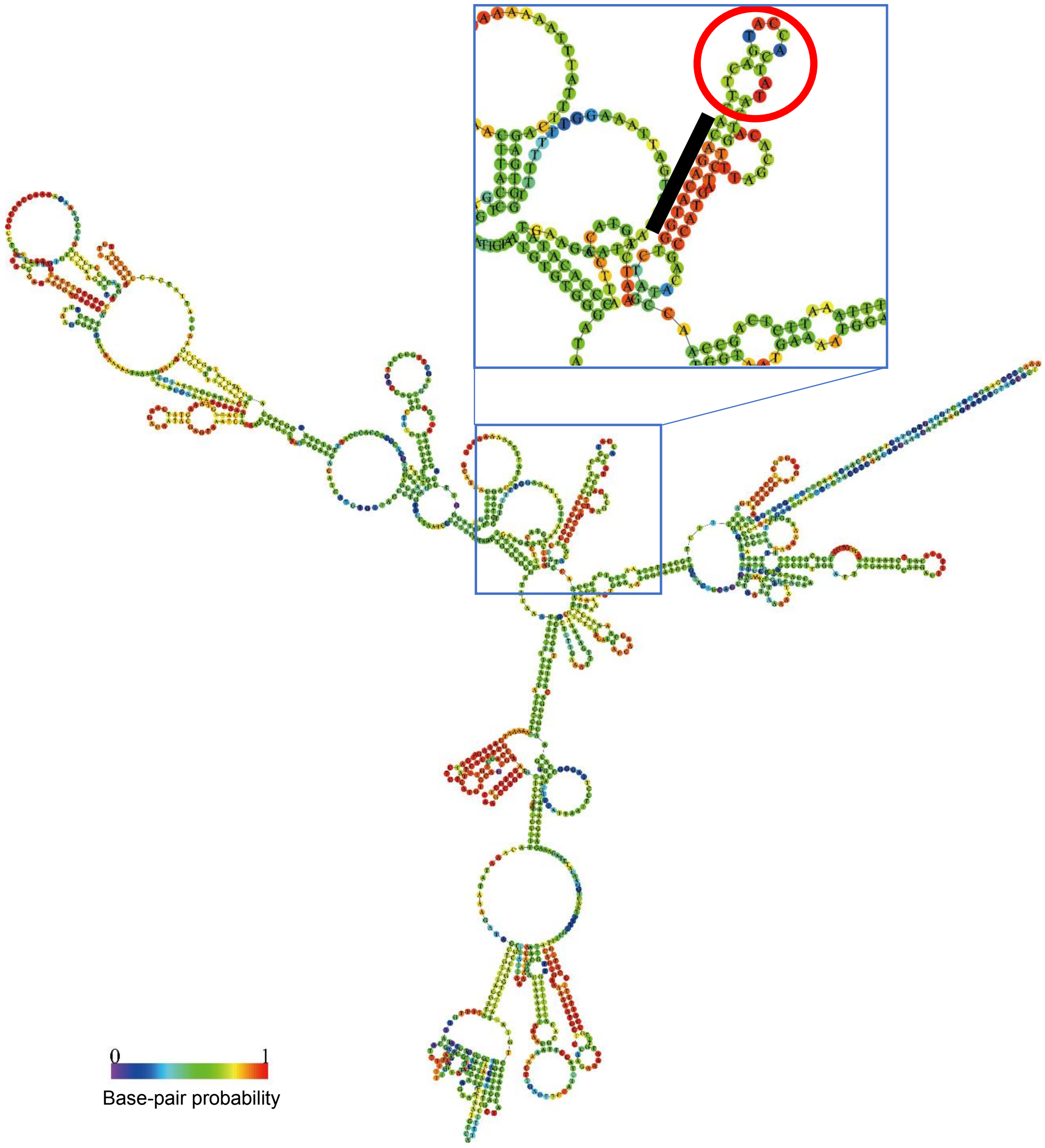

Predicted conformation of the GTRAP3-18 3'-UTR

A schematic representation of the conformation of the GTRAP3-18 3'-UTR containing predicted NOVA1 binding site (red circle) and a miRNA target site (black line) as predicted by Vienna RNAfold software (RNAfold Web Server; http://rna.tbi.univie.ac.at/cgi-bin/RNAWebSuite/RNAfold.cgi). Base-pair probability is indicated in the legend.

Supplementary Figure 12

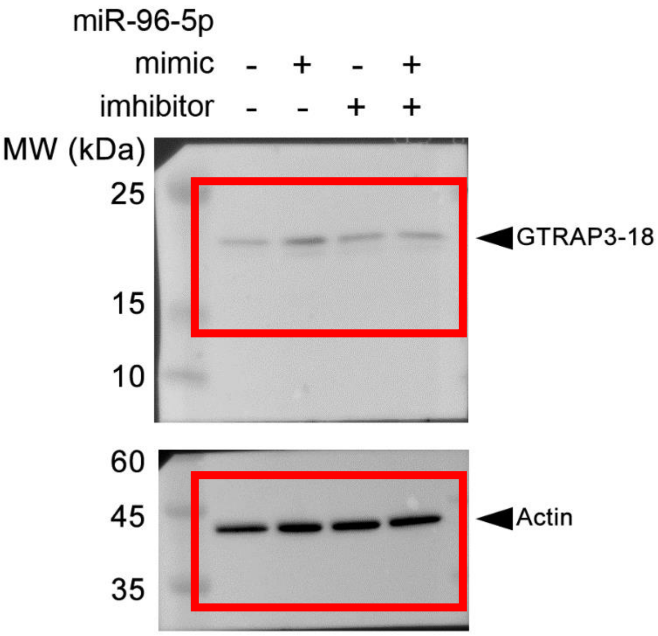

Original blots presented in Fig. 1c

Merged images of full blots and blotting membrane with pre-stained molecular weight marker are shown. Molecular weight is indicated on the left. Red squares indicate cropped sections.

Supplementary Figure 13

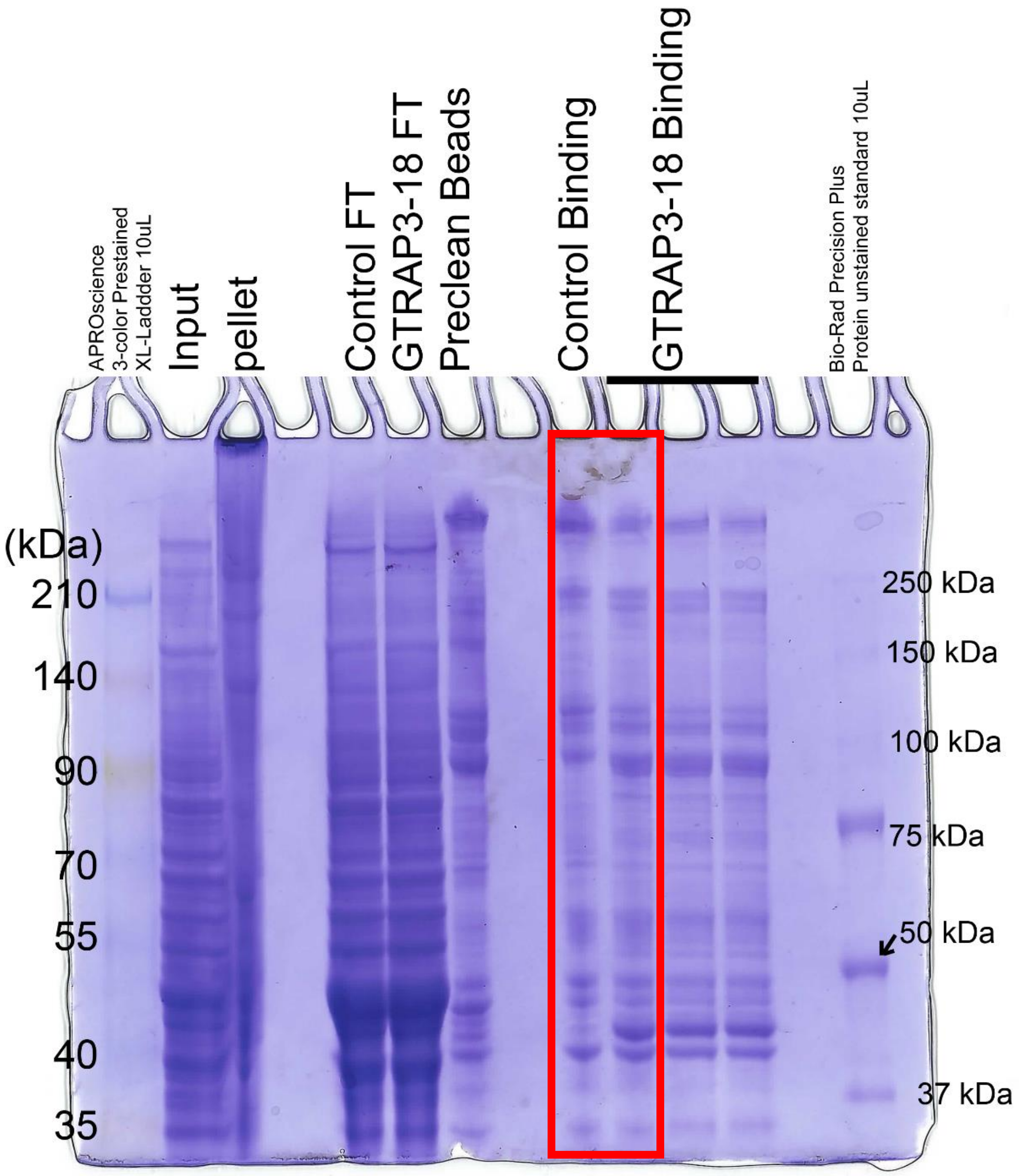

Original CBB-stained gel presented in Fig. 2b

The whole gel which was stained with CBB is shown. Molecular weight markers are indicated on the right and left side of the gel. Red squares indicate cropped sections

Supplementary Figure 14

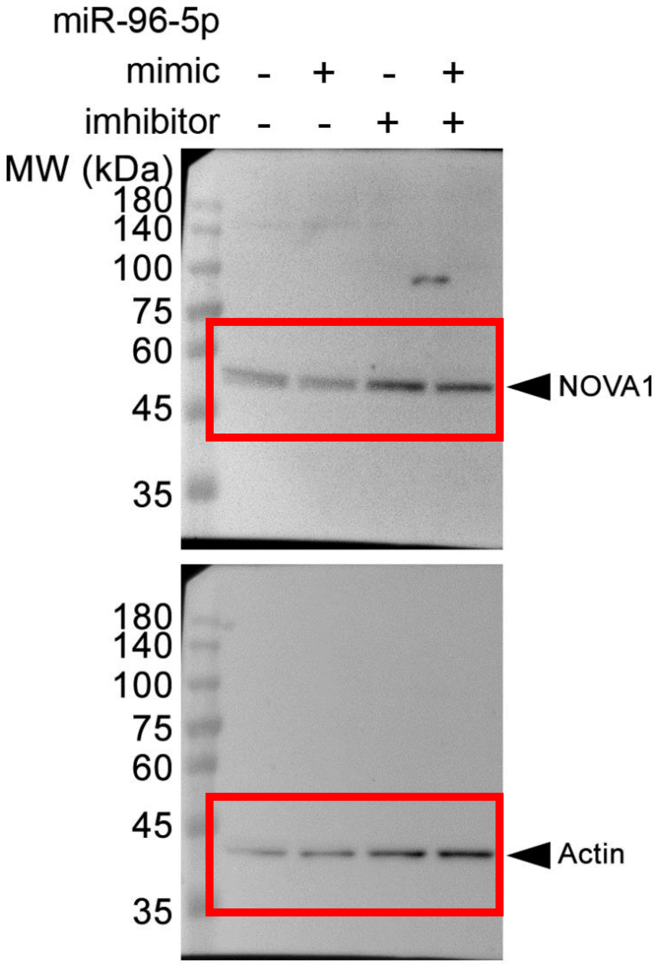

Original blots presented in Fig. 3a

Merged images of full blots and blotting membrane with pre-stained molecular weight marker are shown. Molecular weight is indicated on the left. Red squares indicate cropped sections.

Supplementary Figure 15

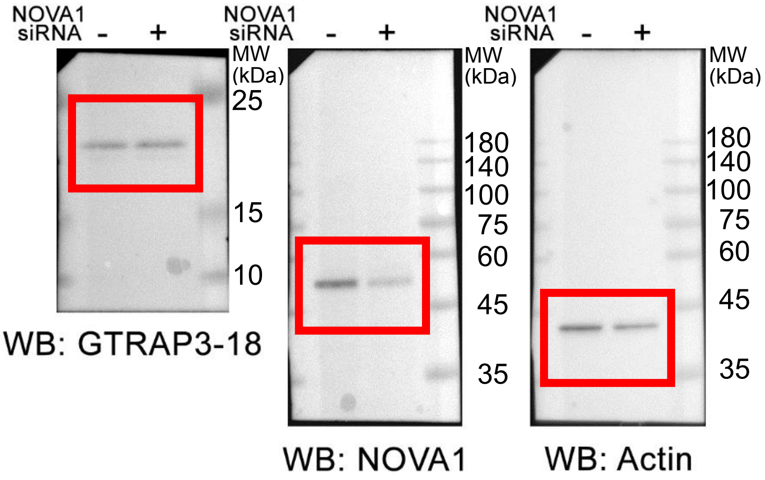

Original blots presented in Fig. 4a

Merged images of full blots and blotting membrane with pre-stained molecular weight marker are shown. Molecular weight is indicated on the right of the blots. Red squares indicate cropped sections.
