# Supplementary material for: Inhibition of miR-96-5p in the mouse brain increases glutathione levels by altering NOVA1 expression
Source: Commun Biol. 2021 Feb 10;4:182. doi: 10.1038/s42003-021-01706-0 (PMC7876013; doi:10.1038/s42003-021-01706-0)
Supplement: Supplementary file 9 — Reporting Summary [file 42003_2021_1706_MOESM9_ESM.pdf]

nature research

Corresponding author(s): Toshio Nakaki

Last updated by author(s): Dec 25, 2020

Reporting Summary

Nature Research wishes to improve the reproducibility of the work that we publish. This form provides structure for consistency and transparency in reporting. For further information on Nature Research policies, see Authors & Referees and the Editorial Policy Checklist .

Statistics

For all statistical analyses, confirm that the following items are present in the figure legend, table legend, main text, or Methods section.

n/a	Confirmed	
☐	☒	The exact sample size (*n*) for each experimental group/condition, given as a discrete number and unit of measurement
☐	☒	A statement on whether measurements were taken from distinct samples or whether the same sample was measured repeatedly
☐	☒	The statistical test(s) used AND whether they are one- or two-sided<br>*Only common tests should be described solely by name; describe more complex techniques in the Methods section.*
☐	☒	A description of all covariates tested
☐	☒	A description of any assumptions or corrections, such as tests of normality and adjustment for multiple comparisons
☐	☒	A full description of the statistical parameters including central tendency (e.g. means) or other basic estimates (e.g. regression coefficient) AND variation (e.g. standard deviation) or associated estimates of uncertainty (e.g. confidence intervals)
☐	☒	For null hypothesis testing, the test statistic (e.g. F, t, r) with confidence intervals, effect sizes, degrees of freedom and P value noted<br>*Give P values as exact values whenever suitable.*
☒	☐	For Bayesian analysis, information on the choice of priors and Markov chain Monte Carlo settings
☒	☐	For hierarchical and complex designs, identification of the appropriate level for tests and full reporting of outcomes
☐	☒	Estimates of effect sizes (e.g. Cohen's d, Pearson's r), indicating how they were calculated

Our web collection on statistics for biologists contains articles on many of the points above.

Software and code

Policy information about availability of computer code

Data collection	Immunoblotting data was collected using Luminograph I (ATTO).<br>Microscopy images were collected using Nikon A1 confocal microscope.<br>Luciferase activity was detected using Luminometer TD-20/20 (Turner Biosystems).<br>LC-MS/MS analysis was performed by Shimadzu techno Research using Q Exactive Plus (Thermo Fisher Scientific).<br>Detection of intracellular ROS level using DCFH-DA assay was performed with DTX 800 Multimode Detector (Beckman Coulter).<br>Quantitative RT-PCR were performed using Applied Biosystems 7500 Fast Real-Time PCR System (Thermo Fisher Scientific)
Data analysis	CS analyzer4 (Ver. 2.2.3) was used for the analysis of protein expression<br>NIS Elements C (Ver. 4.30) was used for the analysis of immunocytochemical and immunohistochemical experiments<br>IBM SPSS Statistics (Rel. 24.0.0.0) was used for statistical analysis (Tukey's HSD test and Students' t-test, two-sided).

For manuscripts utilizing custom algorithms or software that are central to the research but not yet described in published literature, software must be made available to editors/reviewers. We strongly encourage code deposition in a community repository (e.g. GitHub). See the Nature Research guidelines for submitting code & software for further information.

Data

Policy information about availability of data

All manuscripts must include a data availability statement. This statement should provide the following information, where applicable:

- Accession codes, unique identifiers, or web links for publicly available datasets
- A list of figures that have associated raw data
- A description of any restrictions on data availability

All source data for the graphs are listed in Supplementary Data 1-7. Supplementary Data 1 contains the source data underlying Fig. 1, Supplementary Data 2 contains the source data underlying Fig. 3, Supplementary Data 3 contains the source data underlying Fig. 4, Supplementary Data 4 contains the source data underlying Fig. 5, Supplementary Data 5 contains the source data underlying Supplementary Table 1, Supplementary Data 6 contains the source data underlying

October 2018

Supplementary Table 2 and Supplementary Data 7 contains the source data underlying Supplementary Figure 2, 4, 5, 6 and 7. The other datasets generated/ analyzed during the current study are available from the corresponding author (T.K) on reasonable request.

Field-specific reporting

Please select the one below that is the best fit for your research. If you are not sure, read the appropriate sections before making your selection.

☒ Life sciences ☐ Behavioural & social sciences ☐ Ecological, evolutionary & environmental sciences

For a reference copy of the document with all sections, see nature.com/documents/nr-reporting-summary-flat.pdf

Life sciences study design

All studies must disclose on these points even when the disclosure is negative.

Sample size	No statistical method for sample size determination. We chose the sample size for each experiment which is statistically significant when we performed similar experiments in the previous study. In the current study chosen sample size was sufficient to yield clear statistical significance.
Data exclusions	No data was excluded from the analyses.
Replication	All experiments in the current study was repeated at least three independent experiments. All attempts at replication were successful. All experimental procedures are precisely described in the Methods section.
Randomization	No randomization was performed. The results were semi-automatically analyzed by the built-in device software.
Blinding	No blinding was performed because of same reason with "Randomization" section.

Reporting for specific materials, systems and methods

We require information from authors about some types of materials, experimental systems and methods used in many studies. Here, indicate whether each material, system or method listed is relevant to your study. If you are not sure if a list item applies to your research, read the appropriate section before selecting a response.

Materials & experimental systems

n/a	Involved in the study	
☐	☒	Antibodies
☐	☒	Eukaryotic cell lines
☒	☐	Palaeontology
☐	☒	Animals and other organisms
☒	☐	Human research participants
☒	☐	Clinical data

Methods

n/a	Involved in the study	
☒	☐	ChIP-seq
☒	☐	Flow cytometry
☒	☐	MRI-based neuroimaging

Antibodies

Antibodies used	Antibodies used in the current study are anti-GTRAP3-18 (Novus Biologicals; NBP-84273, Lot# A106837), anti-NOVA1 (Abcam; ab183024, Lot# GR3192980-4), anti-Actin (Sigma-Aldrich; A5316, clone AC-74, Lot# 096M4855V), anti-EAAC1 (Abcam; ab124802, Lot# GR155501-2), anti-GTRAP3-18 (Novus Biologicals; NB100-1105, Lot# C2 N011008), anti-NeuN (Millipore; MAB377, Lot# LV1728859), anti-Iba1 (Wako; 019-19741, Lot# WDJ3047), anti-GFAP (Sigma; G-3893, Lot# 063K4851), anti-LC3 (MBL; PM036MS, Lot#004), anti-4-HNE (abcam; ab48506, Lot# GR3251707-2), HRP-labelled secondary antibodies against rabbit IgG (Chemicon; AP188P, Lot# 2976349), HRP-labelled secondary antibodies against mouse IgG (Chemicon; AP181P, Lot# 3096500), Alexa Fluor 546 anti-mouse IgG (Molecular Probes; A11003, Lot# 45705A), Alexa Fluor 594 anti-mouse IgG (Molecular Probes; A11005, Lot# 99E2-1), Alexa-Fluor 647 anti-rabbit IgG (Molecular Probes; A31573, Lot# 1322326), Alexa-Fluor 488 anti-goat IgG (Molecular Probes; A11055, Lot# 1182671), Alexa-Fluor 488 anti-rabbit IgG (Molecular Probes; A11008, Lot# 38649A).
Validation	Validation statements of all antibodies used in the current study were referred to the manufacturer's datasheets.

Eukaryotic cell lines

Policy information about cell lines

Cell line source(s)	SH-SY5Y cell line was obtained from Dr Shinichi Kohsaka, National Institute of Neuroscience in 2002.<br>HEK293 cell line was purchased from DS Pharma Biomedical Co., Ltd.<br>Neuro2a cell line was purchased from KAC Co., Ltd.

Authentication	The cells were authenticated by checking morphology using microscope and growth curve analysis.
Mycoplasma contamination	All cell lines were tested by checking with DAPI staining and were found to be mycoplasma-free.
Commonly misidentified lines (See ICLAC register)	No misidentified lines of cultured cells were used in the current study.

Animals and other organisms

Policy information about studies involving animals; ARRIVE guidelines recommended for reporting animal research

Laboratory animals	Adult male C57Bl/6 mice (8 weeks old) were used in the current study.
Wild animals	The study did not involve wild animals.
Field-collected samples	The study did not involve field-collected samples.
Ethics oversight	All animal protocols were approved by the Animal Experimentation Committee of the Teikyo University School of Medicine.

Note that full information on the approval of the study protocol must also be provided in the manuscript.
