# Supplementary material for: Inhibition of miR-96-5p in the mouse brain increases glutathione levels by altering NOVA1 expression
Source: Commun Biol. 2021 Feb 10;4:182. doi: 10.1038/s42003-021-01706-0 (PMC7876013; doi:10.1038/s42003-021-01706-0)
Supplement: Supplementary file 10 — Description of Supplementary Files [file 42003_2021_1706_MOESM10_ESM.pdf]

Description of Additional Supplementary Files

File name: Supplementary Data 1
Description: Source data underlying Fig. 1.

File name: Supplementary Data 2
Description: Source data underlying Fig. 3.

File name: Supplementary Data 3
Description: Source data underlying Fig. 4.

File name: Supplementary Data 4
Description: Source data underlying Fig. 5

File name: Supplementary Data 5
Description: Source data underlying Supplementary Table 1.

File name: Supplementary Data 6
Description: Source data underlying Supplementary Table 2.

File name: Supplementary Data 7
Description: Source data underlying Supplementary Figure 2, 4, 5, 6 and 7.
